# Standard Reference Materials for Cement Paste, Part I: Suggestion of Constituent Materials Based on Rheological Analysis

**DOI:** 10.3390/ma11040624

**Published:** 2018-04-18

**Authors:** Dong Kyu Lee, Myoung Sung Choi

**Affiliations:** Department of Safety Engineering, Dongguk University-Gyeongju, 123 Dongdae-ro, Gyeongju 38066, Gyeongbuk, Korea; dklee@dongguk.ac.kr

**Keywords:** standard reference materials, flow characteristics, cement paste, rheology, particulate standard materials

## Abstract

The purpose of this study was to develop a standard reference material that can simulate the flow characteristics of cement paste. For this purpose, it is important to determine the constituent materials of the standard material for cement paste. Generally, cement paste is a mixture of cement and water. To determine the constituent material of cement paste, it was divided into powder that can replace cement and matrix fluid. With the concept of rheology, which can evaluate the flow properties of selected materials quantitatively under certain mixing conditions, experiments were carried out step-by-step according to material composition combination, stage of aging, and material types. As a result, limestone powder was determined to be a cement substitute, and glycerol and water were determined to be a matrix fluid substitute. After an analysis of the compatibility with the required properties of the particulate standard materials, the finally selected standard reference material was found to satisfy the required performance.

## 1. Introduction

Most metropolitan areas face various challenges, such as rising land prices, a lack of available land, and limits on the horizontal use of land. Accordingly, the demand for super concrete structures has been increasing constantly, giving birth to several high-rise buildings and large-scale structures [1,2]. On the other hand, with the experience of constructing large concrete structures, and although the construction industry has been recognized for its outstanding construction technology and making remarkable achievements, higher-quality construction technology is still needed for the construction of such super concrete structures. Such high-standard construction technology also requires control of the material properties during construction, having the technology for a quantitative evaluation of a construction material. In short, to be able to analyze the quantitative construction technology, it is essential to analyze materials quantitatively. For this purpose, it is important to develop a reference material that represents consistent characteristics that can be quantified under any condition. This makes it possible to secure more economical and effective construction technology and evaluate the construction performance objectively. Therefore, there is a need to develop a consistent quality control material based on a quantification of the concrete flow performance, which is called a standard reference material [3,4,5,6,7].

The development of a standard reference material enables a stable construction performance evaluation, whose quality can be controlled consistently regardless of the manufacturing process in the stage of construction or the contractor. In addition, the calibration of various rheometers that have already been developed to measure the flow condition and flow performance quantitatively with absolute values, not just examining the initial flow performance evaluation based on a relative comparison, will become possible. Ultimately, the performance in the stages of an initial concrete construction can be evaluated quantitatively, making a scientific pre-construction performance evaluation and utilization of the standard reference material possible. In addition, a standard reference material can also be used in various fields, including use as a recycling sample for pipe circuit pumping tests, which examine pumping performance, a standard reference sample for an evaluation of the replacement cycle test for pump equipment degradation evaluation, and a standard sample for quality control in the field of cutting-edge technology, such as 3D digital printing. On the other hand, concrete is a multicomponent material that contains particles with a wide variety of sizes ranging from minute particles, such as cement, to coarse particles tens of millimeters in diameter [5,6]. Therefore, the development of standard reference materials for the quantitative evaluation of cementitious materials and the substitution of the initial flow characteristics of cement paste have been actively carried out, but there is no clear definition on standard reference materials [7,8,9,10]. The primary objective of this study was to draw constituent materials of the standard reference material for cement paste, which is the most essential component of concrete, based on the concept of rheology that enables an evaluation of the flow properties, for a definition of the standard reference material. To develop standard reference materials for cement paste, the properties required by multicomponent standard reference materials, including particles, should be examined. The requirements for a particle-phase standard reference material currently suggested are as follows: (1) no particle separation during the experiment; (2) presentation of a linear Bingham reaction under a wide shear strain; (3) no change in rheological or chemical property between the fluid and particles over a long period of time; (4) sufficient yield stress to prevent the material separation of the aggregate; and (5) almost no double-sided linear response behavior (hysteresis) [7]. This study examined several tentative materials for particle-phase standard reference materials for cement paste and analyzed their rheological properties under certain mixing conditions. This paper describes the result of an evaluation of materials that are suitable for the properties required by a particle-phase standard reference material. Based on the results of this study, along with calibration materials for various rheometer measurement systems, research on the standard reference material should be expanded step-by-step from minute particles to fine aggregates and then to coarse aggregates, including the standard reference material for mortar all the way to the material for concrete [11,12,13].

## 2. Experimental Plan and Methodology

### 2.1. Experimental Plan

Generally, for cement paste, cement powder and matrix fluid can be considered as two representative components. The flow performance of powder that can replace cement when made in the form of a mixture, as well as a matrix fluid in the form of a mixture of cement and water, should be investigated carefully. Table 1 lists the constituent materials used in this study. Limestone powder, blast furnace slag, silica powder, and meta kaolin that showed little reaction to moist conditions and whose mean grain diameter is similar to that of cement were selected as the tentative substitute for cement powder. Table 2 lists the constituents of each material [14,15,16,17]. In addition, corn syrup and glycerol, which have properties similar to the flow performance properties of cement paste matrix, as well as chemical stability showing a consistent viscosity with time, were selected as the tentative substitute for the matrix fluid [18,19]. Table 3 lists the chemical composition of glycerol. A corn syrup product that includes 100% natural pure corn starch was used. As listed in Table 4, the experiment was carried out stage-by-stage. After reviewing the required properties of the standard reference materials, tentative materials for the standard materials were selected, and rheological analysis incorporating composition combinations, initial mixture conditions, and material types were then investigated. At the end, materials selected for the standard reference materials were evaluated based on requirements for particulate standards. The mixture was prepared by blending glycerol or corn syrup selected as a matrix fluid for each of limestone powder, blast furnace slag, silica powder, and meta kaolin.

### 2.2. Experiment Method

**Figure 1:**
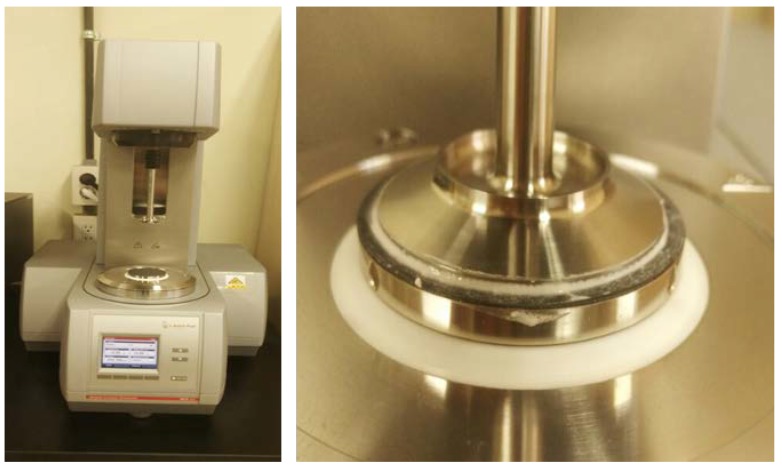
Anton paar Rheometer.

For this study, a rheology experiment that can evaluate the initial flow performance was conducted primarily to examine the properties required for a particle-phase standard reference material. The ingredients were mixed in four steps spanning 120 s (15 s, 15 s, 30 s, and 60 s) using a high-speed mixer. At the end of each step, the ingredients were kneaded for even mixing of the ingredients. The rheological properties were tested at a consistent temperature (20 °C) and time using an Anton Paar Rheometer (Figure 1). Generally, the rheology is determined by the relationship between the shear stress and shear rate that affect the materials. This study used the Bingham model given in Equation (1) to determine the plastic viscosity and yield stress. In this method, the plastic viscosity is defined with the inclination of the shear stress, shear rate, and yield stress as the y-intercept, which is determined by regression analysis.

(1) $$\tau =\eta \dot{\gamma }+\tau_{0}$$

where τ, η, $\dot{\gamma }$, and $\tau_{0}$ are the shear stress, plastic viscosity, shear rate, and yield stress, respectively. Before beginning the experiment, the samples were rotated for 60 s at a shear rate of 50 s^−1^ for the homogenization of all the ingredients and they were then given 10 s rest to reach equilibrium. The shear rate was increased from 0.1 s^−1^ to 40 s^−1^ and then decreased to 0.1 s^−1^, and on the upward and downward curve, the shear resistance put on a spindle by the rotation velocity divided into 10 phases was measured. A serrated spindle, 50 mm in diameter, was used to prevent the separation and slip of the ingredients [20,21,22].

## 3. Result and Analysis

### 3.1. Rheology Analysis According to the Constituent Composition System

**Figure 2:**
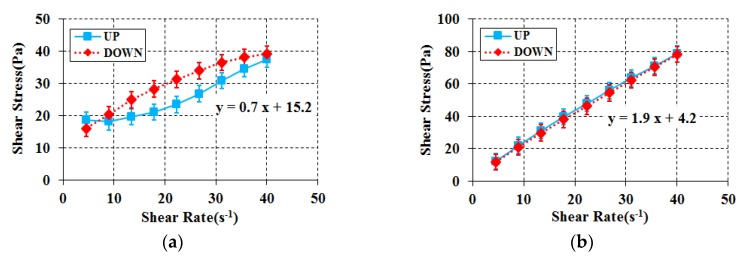
Rheology for component system. (**a**) Limestone powder + Water; (**b**) Limestone powder + Glycerol + Water.

First, the role of each substance of the cement substitute and the matrix fluid substitute was analyzed to determine the constituent mixture composition of the particle-phase standard reference material to be developed. The first question to examine for the constituent composition is whether to make the ingredient composition for determining the standard reference material a two-component composition system or a three-component composition system. For this review, three combinations were investigated as listed in Table 5. Here, limestone powder was used for the cement substitute. Limestone powder was believed to be the most stable for the entire process of the experiment and has a low level of reaction to moisture. In addition, as corn syrup contains water, glycerol, which does not contain water and enables relatively easier control of the composition proportion, was used to improve the accuracy of the experiment. Figure 2 presents the results of the rheology analysis for each combination. The two-component combination system of limestone powder and glycerol was not analyzed in the experiment because it exceeded the capacity of the rheometer’s torque, so it was excluded from the experiment result.

Based on the rheology experiment on each of the component systems, their compatibility with the properties required by the particle-phase standard reference material was analyzed. As shown in Figure 2a, double-sided non-linear response behavior (hysteresis) was detected in the two-component combination system of limestone powder and water. On the other hand, the three-component combination system of limestone powder, glycerol, and water satisfied all of the requirements for the particle-phase standard reference material, without any material separation or hysteresis. This indicates that the use of a matrix fluid (glycerol) plays an important role in preventing material separation and double-sided linear response behavior. Therefore, the three-component composition system is essential for the particle-phase standard reference material to be developed.

### 3.2. Rheology Analysis in Early Stage of Aging

**Figure 3:**
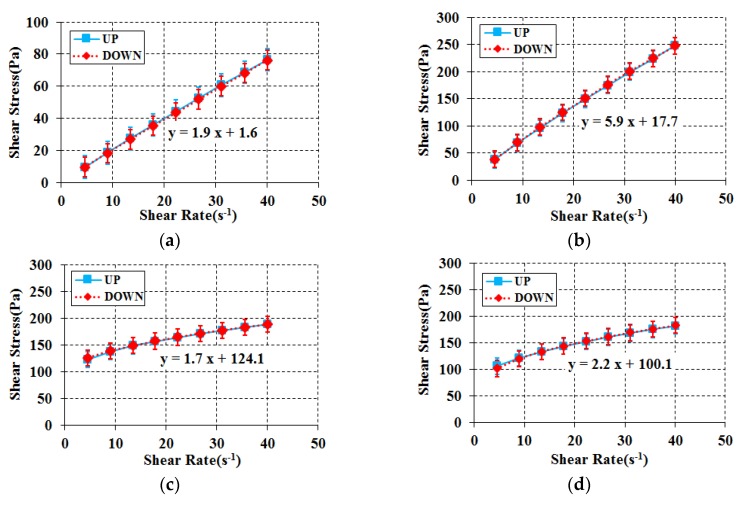

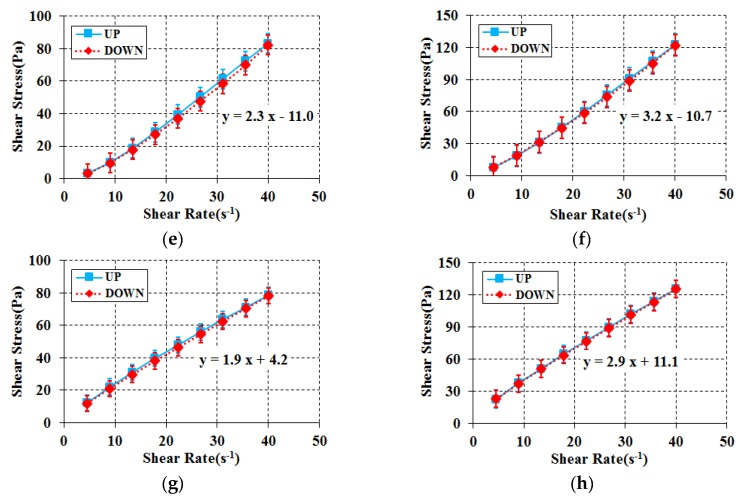
Rheology for all combinations. (**a**) Blast furnace slag + Glycerol + Water; (**b**) Blast furnace slag + Corn syrup + Water; (**c**) Meta kaolin + Glycerol + Water; (**d**) Meta kaolin + Corn syrup + Water; (**e**) Silica powder + Glycerol + Water; (**f**) Silica powder + Corn syrup + Water; (**g**) Limestone powder + Glycerol + Water; (**h**) Limestone powder + Corn syrup + Water.

The flow performance in the early stage of aging was analyzed by mixing each of the selected materials in specific proportions. The objective of this analysis was to evaluate the compatibility with the four required properties: (1) no particle separation during the experiment; (2) presentation of a linear Bingham reaction within a wide shear strain; (3) sufficient yield stress to prevent material separation of the aggregate; and (4) almost no double-sided linear response behavior (hysteresis). Figure 3 shows the results of the experiment for each composition. Shear thinning, which refers to a decrease in plastic viscosity following an increase in shear rate, occurred for all compositions of glycerol and corn syrup in the case of meta kaolin [23,24,25]. In contrast, for silica powder, a shear thickening phenomenon that refers to a rise in plastic viscosity following an increase in shear rate was observed in all compositions, showing a very low yield [26,27,28]. For limestone powder and blast furnace slag, a linear Bingham reaction was observed in the entire range of shear strains of all of the corn syrup and glycerol compositions. The compatibility of each composition in the early stages of aging could be identified considering the four requirements for a particle-phase standard reference material as shown in Table 6. From the results, limestone powder and blast furnace slag were found to be tentative cement substitutes that fulfill all of the requirements.

### 3.3. Time Elapse Analysis

#### 3.3.1. Evaluation of the Cement Substitute

**Figure 4:**
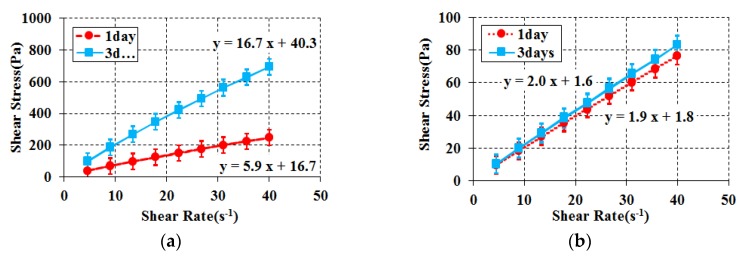
Rheology for elapsed time (Blast furnace Slag case). (**a**) Blast furnace slag + Corn syrup + Water; (**b**) Blast furnace slag + Glycerol + Water.

**Figure 5:**
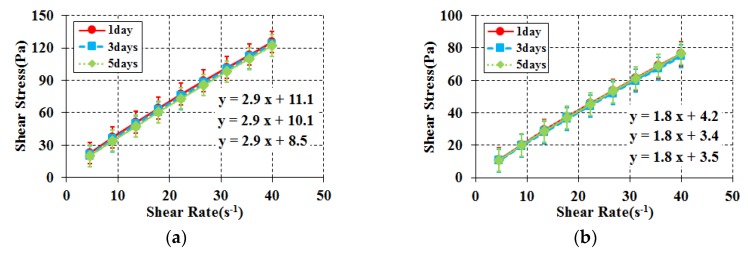
Rheology for elapsed time (Limestone powder case). (**a**) Limestone powder + Corn syrup + Water; (**b**) Limestone powder + Glycerol + Water.

Time elapse rheology analysis was conducted to evaluate rheological and chemical property changes over a longer period, beyond the properties required for a particle-phase standard reference material. This is an important factor that enables an evaluation of the properties of a standard reference material that shows a constant flow performance regardless of time. To conduct time elapse rheology analysis and observe changes in chemical properties, samples were produced after mixing each material and they were sealed and stored at room temperature. First, rheology analysis was conducted for the limestone powder and blast furnace slag combinations selected from the initial aging flow performance analysis with time immediately after mixing on the third day and then on the fifth day as shown in Figure 4 and Figure 5. For all experiments, the samples were analyzed after remixing using a high-speed mixer. Multiple samples were produced to minimize the change in the mixing proportion.

Generally, in the case of blast furnace slag, it is known as a material with potential hydraulic properties and is known to exhibit hydraulic properties in the presence of an alkaline environment and coexistence with cement. Based on these characteristics, blast furnace slag was selected as a substitute for cement powder. As a result of rheological analysis over time, the plastic viscosity on the third day was 3 times higher than that on the first day in the combination with corn syrup. Also, on the fifth day, the blast furnace slag mixture was hardened and measurement was impossible. Glycerol has also been found to have higher plastic viscosity over time. In other words, the blast furnace slag was judged to have a chemical reaction due to its latent hydraulic characteristics over time, and was excluded from the candidates for the substitute for cement powder [29,30,31,32]. On the other hand, limestone powder demonstrated consistent rheological properties regardless of the passage of time, both in its mixture with corn syrup and with glycerol. Limestone powder was eventually selected as the cement substitute based on the result of rheological and chemical property analysis over a long period of time.

#### 3.3.2. Evaluation on Matrix Fluid

**Figure 6:**
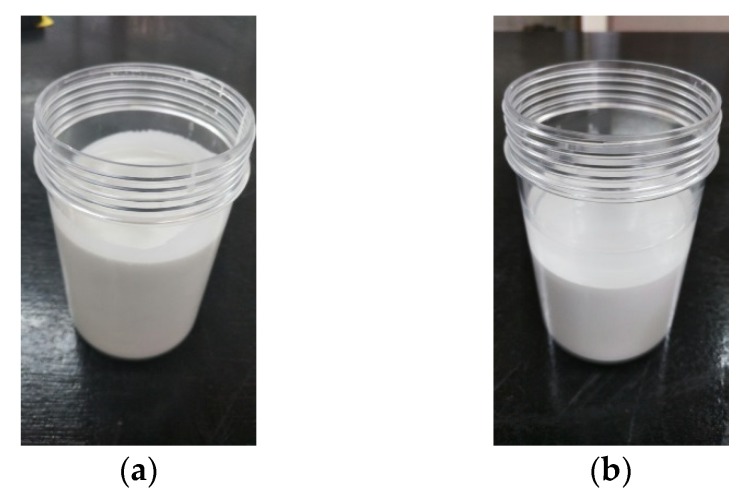
Chemical change of specimen with long-term (Limestone powder + Glycerol + Water). (**a**) Immediately after mixing; (**b**) After 30 days.

**Figure 7:**
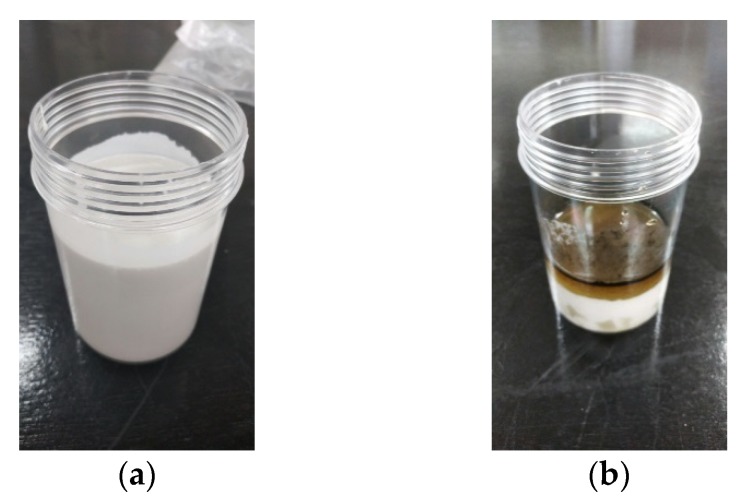
Chemical change of specimen with long-term (Limestone powder + Corn syrup + Water). (**a**) Immediately after mixing; (**b**) After 30 days.

The changes in a sample produced to analyze the chemical properties according to the type of matrix fluid (corn syrup, glycerol) were observed using limestone powder, the selected cement substitute that showed consistent rheological properties with time. As shown in Figure 6 and Figure 7, the mixture with the corn syrup matrix fluid began to show a chemical reaction from approximately two weeks after mixing. On the 30th day, discoloration from mold production and other factors was observed on the surface of the sample. On the other hand, the sample mixed with glycerol showed no change in chemical properties over the 30 days, and the initial flow performance condition was reproduced by carrying out re-mixing. Based on those results, the properties required for a particle-phase standard reference material were evaluated as listed in Table 7. Based on the above results, when the required properties of the particulate standard reference material were evaluated, in the case of limestone powder, a chemical reaction occurred in combination with corn syrup, but all the required characteristics were satisfied in the combination with glycerol. Blast furnace slag reacted chemically in all formulations, such as corn syrup and glycerol. Silica powder did not show a linear Bingham reaction in all formulations, such as corn syrup and glycerol, and low yield stress and a chemical reaction occurred. Meta kaolin also showed no linear Bingham reaction in all formulations, such as corn syrup and glycerol, and a chemical reaction occurred in combination with corn syrup as listed in Table 8. Based on the result of rheological and chemical property analysis, glycerol was eventually selected as the matrix fluid substitute.

### 3.4. Analysis of Selected Constituent Materials by Different Type

**Figure 8:**
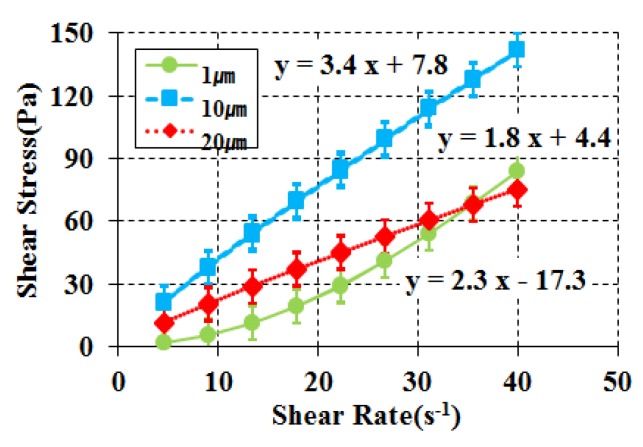
Rheology for different sizes of Limestone powder.

**Figure 9:**
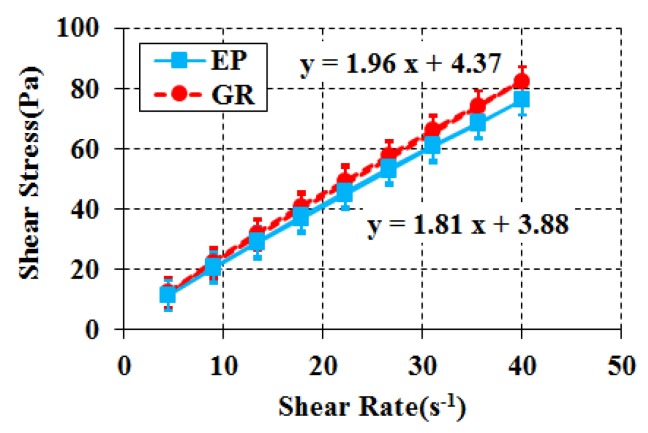
Rheology for different Glycerol grades (EP and GR).

A limestone powder, glycerol, and water combination was found to be the most suitable for the properties required for a particle-phase standard reference material of cement paste. The particle-phase standard reference material to be developed should have consistent flow performance within the error range, and for such a purpose, the particle size of the limestone powder and the grade of the glycerol need to be considered. For all of the abovementioned experiments, Extra Pure (EP) grade glycerol and limestone powder with a 20 μm grain diameter were used. Currently, three types of limestone powder with 1 μm, 10 μm, and 20 μm particle sizes are manufactured at factories, while glycerol is produced in two grades: Extra Pure (EP) grade and Guaranteed Reagent (GR) grade. Also, rheology analysis was conducted according to the particle size of the limestone powder (1 μm, 10 μm, and 20 μm) and the glycerol grade (EP and GR), as shown in Table 8, to determine the changes in the flow performance of each type. For comparison with other experiments, EP-grade glycerol was used for the analysis according to the limestone powder particle size and limestone powder with a 20 μm particle diameter was used for the experiment by glycerol grade. The results are shown in Figure 8 and Figure 9. In the experiment according to the limestone powder particle size, the 1 μm particle size showed the shear thickening phenomenon of an increasing plastic viscosity with increasing shear rate. These results suggest that the smaller the particle size, i.e., the higher the concentration, the greater the occurrence of shear thickening. It is believed that the smaller the particle size, the higher the plastic viscosity due to the increase in interfacial friction area [10]. For 10 μm and 20 μm particle size limestone powder, all of the requirements for a particle-phase standard reference material were satisfied, but the plastic viscosity increased with decreasing particle size, which was attributed to the increasing friction area among particles with decreasing particle size [7]. The plastic viscosity was slightly higher for GR than EP in the experiment using different glycerol grades, but both types of glycerol fulfilled the requirements for a particle-phase standard reference material. Overall, when considering the average particle size of cement, the results recommend the use of limestone powder with a 20 μm particle size and either glycerol grade.

## 4. Conclusions

This study aimed to develop a particle-phase standard reference material that can simulate the flow performance of cement paste, the most primary component of concrete. Three constituents, a cement substitute, a matrix fluid substitute, and water, were selected for the composition of the standard reference material to be developed. Considering their physical and chemical properties, limestone powder, blast furnace slag, meta kaolin, and silica powder, which undergo almost no reaction with water, were selected as the cement substitutes. Corn syrup and glycerol, which have a consistent viscosity over time and chemical stability, were selected as the matrix fluids. In the experiment and analysis, compatibility with the properties required for a particle-phase standard reference material considering the mixture composition systems (two-component system, three-component system), stage of aging, time elapse, and material types (particle sizes and grades) was analyzed to obtain the final composition of the standard reference material for cement paste. The results can be summarized as follows:

(1) In rheology analysis of the two-component composition system with limestone powder and water, double-sided non-linear response behavior was observed. On the other hand, the three-component composition system with limestone powder, glycerol, and water satisfied all of the requirements for a particle-phase standard reference material. Therefore, a three-component composition was found to be appropriate as a standard reference material for cement paste.
(2) In rheology analysis of the initial aging stages of each mixture of cement substitute, shear thinning occurred for meta kaolin, and shear thickening occurred for silica powder. Both limestone powder and blast furnace slag were found to fulfill the requirements for the initial aging stage of the particle-phase standard reference material.
(3) Changes in the flow performance and chemical properties with time were analyzed. The plastic viscosity increased with time elapse in the blast furnace slag, and the strength was rendered in all samples. However, consistent flow performance and chemical properties with time were obtained with limestone powder, which satisfied all of the properties required for a particle-phase standard reference material.
(4) In a sample mixed with corn syrup matrix fluid, a chemical reaction, including mold production and discoloration, occurred. The sample mixed with glycerol exhibited chemical stability with time.
(5) Rheology analysis was conducted to examine the changes in flow characteristics by the particle sizes of limestone powder and grades of glycerol. The result showed that the plastic viscosity increased with a decreasing particle size of limestone powder, and all of its samples except for the one with a 1 μm particle size fulfilled the properties required for a particle-phase standard reference material. All grades of glycerol satisfied the requirements for a particle-phase standard reference material.
(6) The composition of a standard reference material for cement paste, the primary component of concrete, was examined and the combination of limestone powder as a cement substitute, glycerol as a matrix fluid substitute, and water was found to be the most suitable. Further research to propose constituent compositions of standard reference materials of mortar and concrete will be needed based on the mixture combination suggested by this study.

## Figures and Tables

**Table 1:** Tentative substitution of materials for standard reference material (SRM).

Cement Substitute	Matrix Fluid Substitute	Water
Limestone powder	Corn syrup Glycerol	Distilled Water
Blast furnace slag		
Silica powder		
Meta kaolin		

**Table 2:** Analysis of constituent components of cement substitution materials.

Cement Substitute	Constituent (%)								
	SiO_2_	Al_2_O_3_	Fe_2_O_3_	MgO	CaO	K_2_O	Na_2_O	SO_3_	TiO_2_
**Limestone Powder**	0.30	0.10	0.02	0.20	99.30	-	-	-	-
**Blast Furnace Slag**	34.69	14.31	0.50	3.93	41.95	-	-	2.61	-
**Silica Powder**	99.50	0.40	0.05	0.02	0.02	0.02	0.02	-	0.05
**Meta Kaolin**	53	44	0.25	0.22	0.40	0.23		-	-

**Table 3:** Analysis of glycerol components.

Matrix Fluid Substitute	Constituent (%)								
	Contents	NH_4_	SO_4_	As	Fe	Pb	Acid-Base	Fatty Acid Ester	Sulfate
**Glycerol**	99.0	0.005	0.002	0.0002	0.0003	0.0004	0.005	0.2	0.015

**Table 4:** Steps for SRM development.

Step	Contents
**1 Step**	Review particulate standards requirements
**2 Step**	Selection of tentative materials
**3 Step**	· Composition combination · Initial mixture analysis · Analysis by time · Analysis by type (particle sizes and grades)
**4 Step**	Final review of SRM components

**Table 5:** Component system for SRM.

Composition	Cement Substitute	Matrix Fluid Substitute	Water
**2-Component system**	Limestone powder	-	Distilled Water
	Limestone powder	Glycerol	-
**3-Component system**	Limestone powder	Glycerol	Distilled Water

**Table 6:** Evaluation for the required rheology properties for all combinations.

Item			Separating Resistance	Linearity	Yield Value	Hysteresis
**Distilled Water**	**Limestone powder**	**Corn syrup**	O ^1^	O	O	O
		**Glycerol**	O	O	O	O
	**Blast furnace slag**	**Corn syrup**	O	O	O	O
		**Glycerol**	O	O	O	O
	**Silica powder**	**Corn syrup**	O	X ^2^	X	O
		**Glycerol**	O	X	X	O
	**Meta kaolin**	**Corn syrup**	O	X	O	O
		**Glycerol**	O	X	O	O

^1^ indicates conformity; ^2^ indicates unconformity.

**Table 7:** Assessment of conformity for all combinations.

Item			Separating Resistance	Linearity	Yield Value	Hysteresis	Chemical Stability
**Distilled Water**	**Limestone powder**	**Corn syrup**	O	O	O	O	X
		**Glycerol**	O	O	O	O	O
	**Blast furnace slag**	**Corn syrup**	O	O	O	O	X
		**Glycerol**	O	O	O	O	X
	**Silica powder**	**Corn syrup**	O	X	X	O	X
		**Glycerol**	O	X	X	O	X
	**Meta kaolin**	**Corn syrup**	O	X	O	O	X
		**Glycerol**	O	X	O	O	O

**Table 8:** Experiment for different type of limestone and glycerol.

Type	Limestone Powder	Glycerol	Water
Limestone powder particle size experiment	1 µm	Grade (EP ^1^)	Distilled Water
	10 µm		
	20 µm		
Glycerol Experiment by grade	20 µm	Grade (EP)	
		Grade (GR ^2^)	

^1^ EP = Extra Pure; ^2^ GR = Guaranteed Reagent.
